# Pre-emptive Allogeneic Hematopoietic Stem Cell Transplantation in Ataxia Telangiectasia

**DOI:** 10.3389/fimmu.2018.02495

**Published:** 2018-10-29

**Authors:** Shahrzad Bakhtiar, Sandra Woelke, Sabine Huenecke, Matthias Kieslich, Alexander Malcolm Taylor, Ralf Schubert, Stefan Zielen, Peter Bader

**Affiliations:** ^1^Division for Stem Cell Transplantation and Immunology, Children's Hospital, Goethe-Universität Frankfurt am Main, Frankfurt, Germany; ^2^Department of Allergology, Pneumology and Cystic Fibrosis, Children's Hospital, Goethe-Universität Frankfurt am Main, Frankfurt, Germany; ^3^Department of Neuropaediatrics, Children's Hospital, Goethe-Universität Frankfurt am Main, Frankfurt, Germany; ^4^Institute of Cancer and Genomic Sciences, University of Birmingham, Birmingham, United Kingdom

**Keywords:** ataxia telangiectasia, combined immunodeficiency, pre-emptive allogeneic hematopoietic stem cell transplantation, malignancy, primary immunodeficiency

## Abstract

Ataxia telangiectasia (A-T) is a primary immunodeficiency with mutations in the gene encoding the A-T mutated (ATM) protein that interacts with immune, hematopoietic, and endocrine targets resulting in broad multi-systemic clinical manifestations with a devastating outcome. Apart from a progressive neurodegenerative disorder, A-T leads to significantly increased susceptibility to malignancies. It is a matter of discussion whether pre-emptive allogeneic hematopoietic stem cell transplantation (alloHSCT) using a reduced intensity conditioning regimen would be an option to restore immune-competence and prevent malignancy, as shown in animal models, because conventional treatment protocols of malignant diseases using radio- and/or chemotherapy have a high rate of therapy-related morbidity and mortality in these patients. We present the course of the disease, including immune reconstitution and neurological outcome following pre-emptive alloHSCT in a 4-year-old boy with A-T on a 6 year follow-up. Our manuscript provides a proof-of-concept of alloHSCT as an individual pre-emptive treatment strategy from which some A-T patients might benefit.

Ataxia telangiectasia (A-T) is a primary immunodeficiency with mutations in the gene encoding the A-T mutated (ATM) protein that interacts with immune, hematopoietic, and endocrine targets resulting in broad multi-systemic clinical manifestations. Beside a progressive neurodegenerative course, A-T leads to significantly increased susceptibility to malignancies which affects 25% of patients at a median age of 12.5 years (1). It is the subject of ongoing studies to determine whether a lack of immunological surveillance is responsible for the increased risk of malignancy, the disturbed cell regulative capacity of the ATM protein, or both. The incidence of cancer does not correlate with the type of ATM mutation, but rather with the extent of immunodeficiency, particularly profound IgA deficiency and a low number of B cells (1).

In our A-T cohort of 70 patients, we observed malignancies in 16 cases who received chemotherapy by protocol or individualized treatment. Other than three patients under current treatment, 12 others died regardless of the treatment intensity (unpublished data). These results emphasize the substantial need for novel preventive and curative treatment options for malignancies in A-T.

Regarding neurological outcome, a phase III trial is ongoing to assess the effects of monthly transfusions of dexamethasone-loaded autologous erythrocytes, following a phase II trial showing improvement of neurological symptoms (2). The extent to which steroid treatment might have an impact on immunodeficiency and susceptibility to malignancies in A-T patients remains to be evaluated.

Allogeneic hematopoietic stem cell transplantation (alloHSCT), as performed for other genetic instability syndromes, is an encouraging approach to correct immunity and prevent the development of hematologic malignancies. However, alloHSCT is not performed routinely in A-T patients due to the toxicity of the conventional conditioning regimen (3). Herein, we present the course of the disease, including immune reconstitution and neurological outcome following pre-emptive alloHSCT, as an individual treatment strategy to restore immunodeficiency and prevent malignancy, in a 4-year-old boy with A-T on a 6 year follow-up.

## Patient characteristics

The son of non-consanguineous parents of Polish decent presented with upper respiratory infections at the age of 3 years. On immunology work-up very low naïve T cells, an absence of IgA and low IgG2 and IgG4, and an alpha fetoprotein (AFP) level of 52 ng/mL (normal range < 7) were found. Total serum IgG and IgM were in normal range. While patient's previous neurological development and the achievement of the neurodevelopmental steps (sitting at 7 months, walking at 12 months, bi-lingual speech with 5-word sentences at 2 and half years) were normal, he was found to have intermittent gait instability and frequent falls during the second year of life. Additionally multiple telangiectases appeared on skin and conjunctiva. The clinical symptom complex resulted in the diagnosis of A-T, which was confirmed by detecting a compound heterozygous mutation in the ATM gene consisting of two frameshift mutations (c.478_482delTCTCA, p.Ser160Alafs, and c.320delC, p.Pro1069Leufs) causing a complete lack of the ATM protein. Using the Klockgether ataxia score (4), a seven item ataxia scale with a maximum of 35 points (most severe), comparable with the SARA Scale (5), he showed a mild impairment with 2/35 points (gait ataxia 1 point, standing ataxia 1 point). At that stage there was no ataxia of head or extremities, no dysdiadochokinesia, no intention tremor and no dysarthria. Over the course of the disease patient developed a swelling of the proximal interphalangeal joint of his middle finger and small skin lesions at his elbow; being histologically confirmed as granulomas. At the time of transplantation, patient could not be included in the study using steroid loaded erythrocyte transfusions as this study was previously not available in Germany. Written informed consent was obtained from patient's parents for this case report.

## Allohsct and outcome

**Figure 1:**
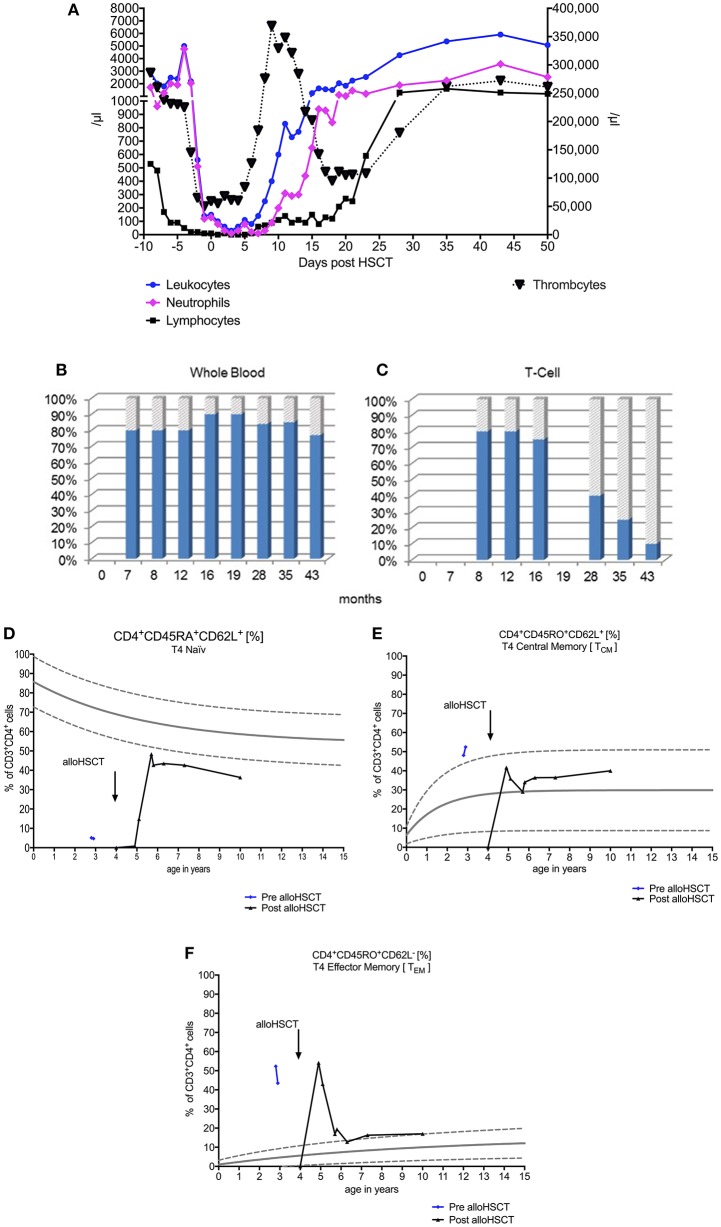
Course of early and stable hematopoietic engraftment after allogeneic hematopoietic stem cell transplantation (alloHSCT) for leukocytes, lymphocytes, and thrombocytes **(A)**. Chimerism analyses show a 10–20% donor origin (donor: shaded gray, recipient: solid blue) in the patient's whole blood **(B)** and that T-cell chimerism significantly increases up to 90% donor **(C)**. The course of immunological reconstitution after alloHSCT shows an expansion of naïve **(D)**, central **(E)**, and effector memory **(F)** CD4^+^ T-cells. Age specific norm values are shown as curves of lower 5%, midline 50%, and upper 95% (6).

**Figure 2:**
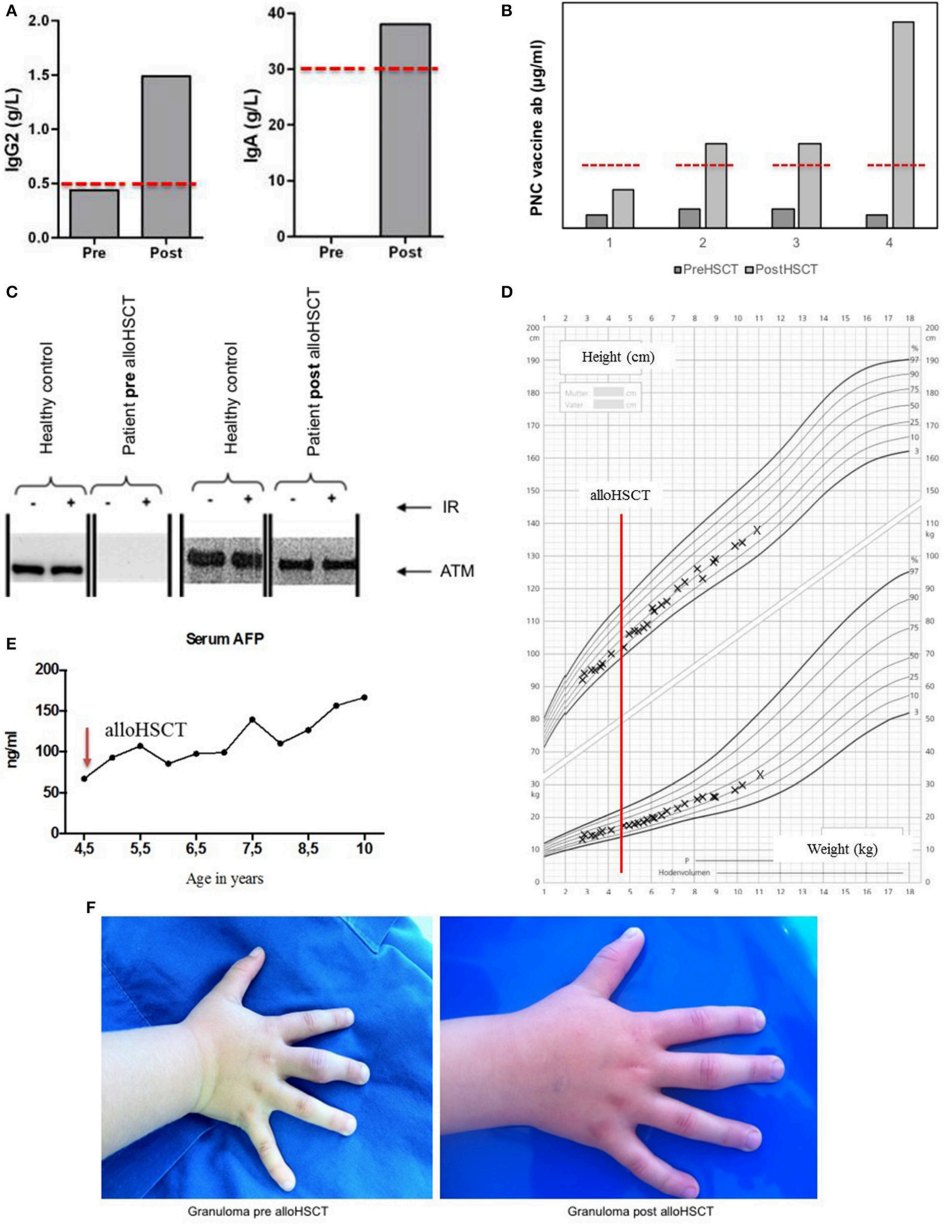
After allogeneic hematopoietic stem cell transplantation (alloHSCT), normal levels (red dashed lines) of IgA and IgG2 **(A)** as well as pneumococcal vaccine antibodies **(B)** are evident. Western blots show normal post-alloHSCT levels of the ataxia telangiectasia mutated (ATM) protein in this patient compared to healthy donors with and without irradiation (IR) of cells in peripheral blood, while having no ATM protein pre-treatment. Lines indicate that the image was cut **(C)**. Patients weight and height prior and after alloHSCT **(D)**. Time course of serum alpha fetoprotein (AFP) levels which are slowly increasing **(E)**. Joint granulomas of this A-T patient resolved completely following alloHSCT **(F)**.

A reduced intensity conditioning (RIC) regimen was used including fludarabine (5 × 30 mg/m^2^/d), cyclophosphamide (4 × 20 mg/kg/d), and rabbit anti-thymocyte globulin (ATG Fresenius® 20 mg/kg/d) on days −4 to −1. Unmanipulated bone marrow of an HLA-10/10 identical sibling was the source of stem cells (5.92 × 10^6^ CD34^+^ cells/kg and 2.96 × 10^7^ CD3^+^ cells/kg). Rapid and stable engraftment was observed by day +15 (Figure 1A). We observed initially a mixed donor chimerism in patient's whole blood (10–20% donor), whereas the patient's T-cells (CD3^+^) reached over 90% of donor origin over time (Figures 1B,C). AlloHSCT corrected the T-cell lymphopenia by expansion of naïve and memory CD4^+^ T-cells, CD19^+^ cells, and CD8^+^ T-cells (Figures 1D–F). Post-alloHSCT, there was an increase in serum immunoglobulins, particularly IgA and IgG2G, to normal levels, as well as in pneumococcal vaccine antibodies (Figures 2A,B). Assessment of the patient's ATM protein showed its complete renewal in peripheral blood cells (Figure 2C). On long term follow up, the patient gained height and weight (Figure 2D). The patient's serum AFP decreased post-alloHSCT and began to increase slowly during subsequent years up to 178 ng/mL at his last follow-up (Figure 2E). There was complete remission of skin and joint granulomas (Figure 2F).

Regarding neurological symptoms, the patient exhibited a milder progression of ataxic symptoms during the 6 year follow-up compared to age-matched A-T patients. On Klockgether score he showed an increase of neurological impairment up to 14 points at 9 years of age (pre-transplant 4 points). Eleven age-matched classical A-T patients between 8 and 10 years of age revealed a median of 19 points (unpublished own data).

## Discussion

Successful engraftment that corrected the immune system and prevented lymphoma in *Atm*-deficient mice using RIC has been shown (7). Moreover, prolonged survival and neurologic improvement of *Atm*-deficient mice was observed through the migration of ATM-competent cells after treatment by syngeneic HSCT (8). Only a few case reports are available on A-T patients undergoing successful alloHSCT as part of their malignancy treatment protocol (9, 10). Nevertheless, undiagnosed A-T patients receiving conventional conditioning regimen can develop severe complications based on their chromosomal instability (11). A recent survey on chromosomal instability syndromes by the European Society of Bone Marrow Transplantation included eight A-T patients with a poor overall survival of 25% and a median follow-up of 35 months (3). Pre-emptive transplantation was not performed in any of these cases. In light of the poor overall survival of transplanted A-T patients, it is a matter of discussion whether pre-emptive alloHSCT using RIC is an option to correct immune-competence and prevent malignancy because conventional treatment protocols of malignant diseases using radio- and/or chemotherapy have a high rate of therapy-related morbidity and mortality in these patients. Novel options, such as anti-cKit antibodies depleting the bone marrow niche, and replacing conventional chemo-based conditioning regimens, might bring a breakthrough in the near future.

In this patient, we observed an event-free course after transplantation without signs of acute toxicity. Furthermore, this treatment provided complete immunological reconstitution and remission of the patient's granulomas without any need for immunosuppression or immunoglobulin replacement. The procedure of alloHSCT did not affect this patient's growth negatively; moreover, we observed an acceptable growth between 10th and 25th percentile, whereas most of the A-T patients between 2–9 years and 10–16 years of age are usually below the 10th and 3rd percentile, respectively (12).

Serum AFP is a reliable marker for A-T patients beyond the second year of life, however its detailed mechanism and the interaction with the ATM protein is not entirely understood yet. AFP levels have been shown to be increasing over time with patients' age and are known to be of hepatic origin (13). Some A-T patients develop a liver disease with increasing liver enzymes. Both Purkinje cells of cerebellum and hepatocytes are rich in mitochondria and sensitive to oxidative stress causing DNA damage. This might provide a common mechanism of neurological damage and increase of AFP over the course of the disease. A link between increasing AFP levels and the degree of patients' clinical neurological impairment was suggested previously (14), however two other studies in recent years could not confirm this finding (15, 16). Further studies are necessary to unravel the underlying mechanism of AFP pathology in larger A-T cohorts.

We observed a slow increase of AFP levels, without any alteration of liver enzymes, post-HSCT in our patient (15). Given the fact that a successful alloHSCT can restore the hematopoietic compartment and might prevent malignancy, an ongoing cell damage in other tissues including AFP-producing tissue, might be expected. Also in presence of mixed donor chimerism, there are residual ATM-deficient cells remaining. Currently, there is a mild progression of neurological impairment in our patient compared to an age-matched A-T cohort. Further clinical follow-up of this patient is necessary during next years including measurement of AFP levels and neurodevelopmental assessment using Ataxia score to investigate whether AFP levels correlate with a progressive neurological impairment.

In conclusion, pre-emptive alloHSCT can correct immunodeficiency in A-T with an acceptable risk of transplant-related mortality, and might be an early treatment of choice in some A-T patients at high risk of hematological malignancy. To what extent the restored immune system and the increase of ATM protein in this patient could prevent the development of other malignancies needs to be evaluated further.

## Author contributions

SB, SW, PB, MK, and SZ provided clinical data, designed the study and wrote the manuscript. AT, RS, and SH provided laboratory data.

### Conflict of interest statement

The authors declare that the research was conducted in the absence of any commercial or financial relationships that could be construed as a potential conflict of interest.
